# Laser Patterning a Graphene Layer on a Ceramic Substrate for Sensor Applications

**DOI:** 10.3390/s20072134

**Published:** 2020-04-10

**Authors:** Marcin Lebioda, Ryszard Pawlak, Witold Szymański, Witold Kaczorowski, Agata Jeziorna

**Affiliations:** 1 Institute of Electrical Engineering Systems, Lodz University of Technology, 90-924 Lodz, Poland; ryszard.pawlak@p.lodz.pl; 2 Institute of Materials Science and Engineering, Lodz University of Technology, 90-924 Lodz, Poland; witold.szymanski@p.lodz.pl (W.S.); witold.kaczorowski@p.lodz.pl (W.K.); agata.jeziorna@p.lodz.pl (A.J.)

**Keywords:** graphene, laser patterning, ceramic substrate, cryogenic

## Abstract

This paper describes a method for patterning the graphene layer and gold electrodes on a ceramic substrate using a Nd:YAG nanosecond fiber laser. The technique enables the processing of both layers and trimming of the sensor parameters. The main aim was to develop a technique for the effective and efficient shaping of both the sensory layer and the metallic electrodes. The laser shaping method is characterized by high speed and very good shape mapping, regardless of the complexity of the processing. Importantly, the technique enables the simultaneous shaping of both the graphene layer and Au electrodes in a direct process that does not require a complex and expensive masking process, and without damaging the ceramic substrate. Our results confirmed the effectiveness of the developed laser technology for shaping a graphene layer and Au electrodes. The ceramic substrate can be used in the construction of various types of sensors operating in a wide temperature range, especially the cryogenic range.

## 1. Introduction

For the last ten years, there has been increasing interest in the use of graphene in electronic devices (all-carbon integrated circuits, transistors), transparent conducting electrodes and circuitry, nanocomposites, supercapacitors, and sensors [1,2,3,4,5,6,7,8,9]. This is due to the extraordinary electronic and thermal properties of graphene, as well as its optical and mechanical potential [9,10,11,12,13]. There are several well-established methods of graphene synthesis, such as mechanical and chemical exfoliation, chemical synthesis, pyrolysis, epitaxial growth, and chemical vapor deposition (CVD) synthesis, but the possibility of graphene patterning at the nano- and microscale is crucial for a wide variety of applications [14,15,16,17]. Various graphene patterning techniques have been developed. Because of the fine 2D structure of graphene, the photolithography process has been applied successfully to the graphene Hall device [18], a graphene anode for an organic light-emitting diode (OLED) [19], interdigitated electrodes for planar micro-supercapacitors [20], and graphene field-effect transistors [21]. However, producing structures using photolithography requires masking and risks chemical contamination, which can cause unintentional doping of the graphene.

Higher pattern resolution is important for producing nanoscale graphene electronic devices. Such structures take advantage of the lateral confinement of electrons within graphene nanoribbons, which introduces an energy gap, and have been achieved by applying ion beam lithography [22,23,24,25], electron beam lithography [26,27,28], or a hybrid process that combines e-beam lithography and helium ion milling [29]. A platinum-coated atomic force microscope tip has been used to induce the locally catalytic reduction of graphene oxide, enabling nanoribbons to be produced with widths of 20–80 nm [30]. Recently, an original method based on reactive inkjet printing was proposed for the production of reduced graphene oxide on textile substrate elements, which were then applied in a supercapacitor [31].

Compared to the graphene patterning techniques outlined above, processes using a laser beam have several advantages, chief among them are high efficiency, short exposure times, low energy consumption, lack of catalysts, repeatability, scalability, and extensive process control capabilities [32,33]. Three groups of laser-based methods for graphene patterning can be distinguished: graphene patterning during synthesis, laser reduction of the graphene oxide (GO), and direct laser writing methods.

Patterned graphene synthesis can be performed using two methods: first, a laser beam is used as an energy source, which is applied directly during the process of laser-induced CVD (LCVD) synthesis; second, the laser beam creates patterns in the catalyst layer, allowing for synthesis in localized areas [32]. Areas of 10 μm^2^ have been patterned using a Nd:YAG continuous-wave (cw)laser (λ = 532 nm) to heat Ni foil in the process of synthesizing graphene from a mixture of CH_4_ and H_2_ [34]. Ribbons of a few layers of graphene have been produced in a CVD process on a polycrystalline Ni wafer that was treated using a cw fiber laser (λ = 1064 nm) [35]. Graphene patterns have been synthesized on glass and SiO_2_ using direct laser writing (λ = 780 nm) in a co-sputtered Ni/C film [36]. Laser-assisted growth and patterning of graphene without a metal catalyst were demonstrated in Wei et al. [37]. A similar approach described in Wei and Xu [38] was used to synthesize arrays of few-layer graphene on Si covered with polymethyl methacrylate (PMMA) using a continuous laser beam (λ = 532 nm). Laser-induced thermal decomposition of the SiC surface (CO_2_ laser, λ = 10.6 μm) [39] allowed for the production of epitaxial graphene in a single-step process. The second method was demonstrated in Kaplas and Svirko [40]. Laser beam ablation or electron-beam lithography was used to create nanostructures on a dielectric substrate (SiO_2_) that was next covered with a thin Cu film in a CVD process. The catalytic film was melted at 700 °C, forming a liquefied Cu network, on which graphene was locally synthesized. 

Various types of lasers have been used for the laser reduction of GO on different substrates. Using a femtosecond laser beam (λ = 790 nm), lines can be produced with widths of about 200 nm [41] or below 2 µm [42]. Femtosecond laser treatment (280 fs, λ = 515 nm) has been applied for the reduction of GO and 3D patterning of lines about 30 µm wide on polymer foil (PET) [43]. When picosecond pulses of 10 ps duration from a Nd:YAG laser with a wavelength of 1064 nm were used, repeated at a frequency of 100 kHz, for graphene oxide reduction, the best effects were achieved by focusing the laser beam on a spot 50 µm in size, which was equal to 50 mW of the mean laser power [44]. Excimer KrF lasers have also been applied for GO reduction and nanopatterning on a micrometer scale of obtained graphene layers [45,46]. Converting GO to reduced GO using a CO_2_ laser has been shown to enable the patterning of supercapacitor devices [47]. Comprehensive studies, which have been performed with three different lasers, have allowed for identifying a mechanism of GO reduction into graphene [48]. This process has a photothermal nature, which explains why the efficiency of GO reduction is higher using nanosecond pulses. The GO reduction requires not only the removal of oxygen atoms, but also transformation to an sp^2^ graphene-like structure, which needs heat. Generally, a single-beam treatment has been applied for the laser reduction of GO and patterning. However, simultaneous reduction and patterning of nanostructures in GO multilayers using the interference of two laser beams with wavelengths of 355 nm and pulse durations of 10 ns has also been performed [49].

In the above-mentioned works [41,42,43,44,45,46,47,48,49], the GO films were prepared via the spin-coating of GO solution. In these processes, the laser-induced reduction of GO had a photothermal character. A new approach for obtaining reduced GO has been recently demonstrated [50,51]. The method involves the local melting of amorphous carbon using a nanosecond laser pulse. During the quenching of melted material, its transformation into reduced GO occurs. Using this nanopatterning method, it was possible to create a p-n junction (n-type rGO and p-type amorphous carbon) [50]. Studies have also shown that the reduced GO film has outstanding ferromagnetic properties [51].

Direct laser patterning of graphene layers is associated with two main problems. On one hand, there is the need to ensure the resolution, fidelity, and dimensional accuracy of the pattern, and on the other, there is the difficulty of completely removing the graphene without damaging the substrate. Achieving these tasks is possible by choosing the appropriate type of laser (femtosecond, picosecond, or nanosecond pulse lasers) and ablation process parameters. Extremely short laser pulses are considered an excellent tool for micromachining graphene layers. Nanometer-scale patterning (width about 400 nm) has been performed using a femtosecond laser (550 fs pulses, λ = 343 nm) and a laser beam converted into a Bessel beam [52]. Using a femtosecond laser (164 fs pulse duration, λ = 780 nm), a large area pattern of microribbons was fabricated in a CVD graphene layer on glass [53]. The high-quality patterning of precise channels in single-layer graphene was obtained using a femtosecond laser (280 fs pulse duration, λ = 1030 nm) [54]. A pulse laser (120 fs pulse duration, λ = 800 nm) allowed for the patterning of graphene and the construction of a working prototype of a flexible Write-Once-Read-Many (WORM) memory card [6]. Precise stripes in graphene, both on SiO_2_/Si and on glass substrates, were patterned using an excimer KrF laser (20 ns pulse duration, λ = 248 nm) [55]. The patterning of lines was achieved using picosecond pulses (30 ps pulse duration, λ = 515 nm) from a fiber-rod-amplified picosecond laser with λ = 515 nm [56]. In the same study, the authors also presented the possibility of the two-photon functionalization (oxidation) of graphene areas with a sub-threshold fluence. The cutting of multilayer graphene has been demonstrated using picosecond laser pulses (15 ps pulse duration, λ = 355 nm) [57]. Interdigitated electrodes were implemented on multilayer graphene films via laser patterning using a nanosecond laser (40 ns pulse duration, λ = 355 nm) [58].

Summarizing the literature and issues discussed above, two conclusions can be made. First, a variety of methods and substrates can be used for graphene synthesis and to support the graphene layer: the chemical vapor deposition of graphene on various materials, such as metallic foil (Cu, Ni) [16,17], SiC [23], SiO_2_/Si [18,20,22], or on glass [19]; transferring CVD graphene onto SiO_2_/Si [21,24,25,52,54], PMMA [26], or on Si/SiN [27]; laser-induced chemical vapor deposition [34,35]; graphene ink coating on glass [57,58]; direct laser synthesis [37,38]; spin-coating a GO solution onto glass [41,42,43,45], polymer [48,49], or on sapphire [30]; and reactive inkjet printing of GO on textile surfaces [31]. Second, different types of lasers can be used for graphene patterning, including nano-, pico-, and femtosecond pulsed lasers generating beams with wavelengths from UV to IR.

Previous studies in the literature relating to graphene on a ceramic substrate have essentially focused on the process of synthesis. Graphene films have been synthesized using a CVD process on Si_3_N_4_ [59], Al_2_O_3_ [60], on a high-κ dielectric substrate like SrTiO_3_ [61], or without a catalyst direct on a ceramic boat [62]. This paper describes a method for patterning the graphene layer and gold electrodes on a ceramic substrate (Al_2_O_3_) using a Nd:YAG nanosecond fiber laser. To the best knowledge of the authors, there are no previous reports regarding the transfer of high-strength metallurgical graphene (HSMG^®^) to a ceramic substrate. The transfer of the graphene layer from a metallic substrate was performed using PMMA as the graphene-supporting material. In terms of using graphene on a ceramic substrate as a sensor layer, it would be very useful to develop a method that allows for graphene to be removed either from ceramics or from an electrode layer (Au), or the graphene and electrode simultaneously. The main goal of the research was to determine the optimal conditions for direct laser recording in these three cases and to examine the efficiency, precision, and purity of the process. The areas of the samples with and without the laser treatment were studied via microscopic examination and Raman spectroscopy. Measurements of the electrical properties of graphene on the ceramic substrate were performed. This enabled the impact of the laser processing on electrical parameters of the samples to be identified via comparison with previous results. The electrical conductivity was measured across a wide temperature range (293–15 K). The results are particularly important for the construction of cryogenic sensors (temperature sensors, Hall sensors, etc.) that are based on graphene.

## 2. Materials and Methods

### 2.1. Materials and Sample Design

We studied samples based on a layer of HSMG^®^ supported on a ceramic substrate with gold electrodes. This configuration enables various types of sensors to be built with HSMG^®^ as the active layer. The ceramic substrate parameters are important for the construction of sensors (resistance temperature detectors (RTDs), Hall sensors, biosensors, etc.) and other devices based on graphene (microheaters, ultrathin electrodes, etc.). All the samples were designed with graphene transferred onto an Al_2_O_3_ ceramic substrate. Alundum ceramics can be used in devices operating over a wide temperature range, including cryogenic temperatures and temperatures over 1300 K. In addition, the ceramic substrate provides very good thermal, mechanical, and dielectric parameters. The thermal expansion of Al_2_O_3_ over a wide temperature range is comparatively small (≈8.2 × 10^−6^ K^−1^) and its thermal conductivity (≈35 W/mK at 293 K) is larger than that of typical polymers or dielectric composite materials. The Al_2_O_3_ ceramic substrate used in this study was extremely pure (>99.5%), with small crystallites (10 μm) and without open porosity. Both surfaces were polished to a roughness value Ra = 0.08 μm. The graphene layer was transferred to the ceramic substrate from a metallic substrate using PMMA as the graphene-supporting material. Before the transfer process, physical vapor deposition (PVD) was used to make flat, gold, nano-thickness (150–200 nm) electrodes on the ceramic surface.

**Figure 1:**
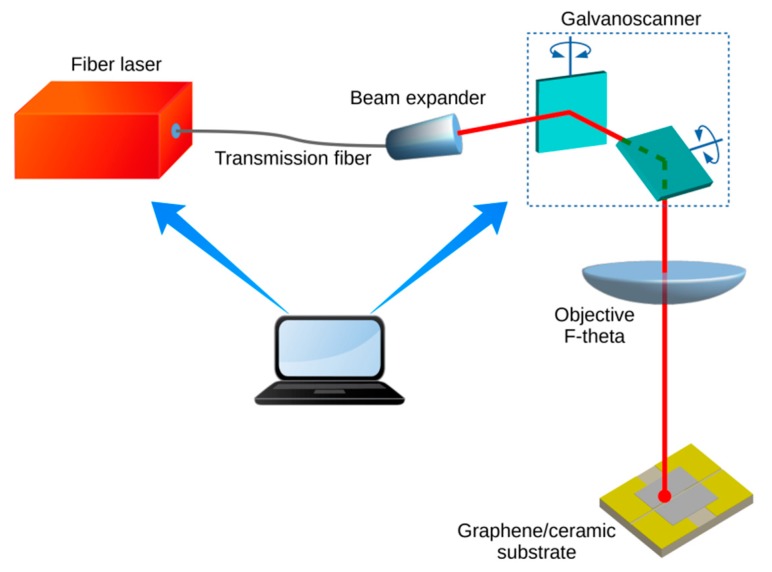
Laser patterning system.

The design of sensors requires high-fidelity mapping of the sensory layer. The precision of this process determines the repeatability of the parameters and the accuracy of the sensor. Laser processing was used to form structures of different geometries and dimensions, both in the HSMG^®^ layer and on the gold nano-thickness electrodes. Figure 1 shows the laser micromachining system used in our studies, consisting of a Nd:YAG fiber laser (SPI G3 SM fiber laser, SPI Lasers UK Ltd., Southampton, UK) with an 8× expander, F-theta lens (GEOMATEC, focal length: 165 mm, GEOMATEC Co., Ltd, Yokohama, Japan), and beam scanner (Xtreme beam scanner, Nutfield Technology Inc., Hudson, NH, USA).

A fiber laser was used to generate a one-mode pulsed beam in the nanosecond range. Shaping of the structures could thereby be performed in a controllable ablation process. When the laser beam was focused on a spot of 26 μm, the Gaussian distribution of the power density provided good accuracy for the ablation process. The experiments were performed using laser pulses of various durations in the range of 15–35 ns, with repetition frequencies from 290 to 80 kHz. The main goal was to remove the graphene layer or the thin gold layer without damaging the ceramic substrate. The pulse energy was therefore finely adjusted according to the pulse duration, repetition frequency, and velocity of beam scanning. The pulse energy varied from 12 μJ to 40 μJ.

**Figure 2:**
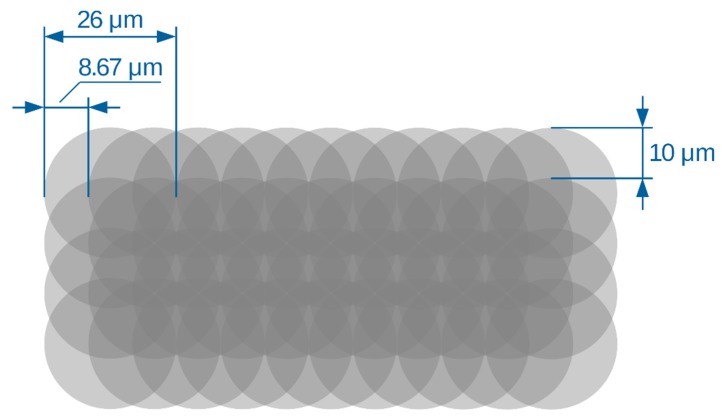
Scheme of the laser patterning procedure.

The same uniform distribution of the laser beam power was obtained independently of the repetition frequency due to an appropriate adjustment of the scanning velocity (Figure 2). A pulse overlap ratio of 1:3 was assumed, which meant, for example, a scanning velocity of 2500 mm/s at a repetition frequency of 290 kHz or 690 mm/s at 80 kHz. Subsequent scan lines were shifted by 10 μm.

To confirm the effectiveness and correctness of the process, samples were tested before and after laser patterning. Only part of each sample was laser treated. The studies included microscopic observations of samples, Raman spectroscopy, and tests of electrical properties over a wide temperature range (15–295 K).

**Figure 3:**
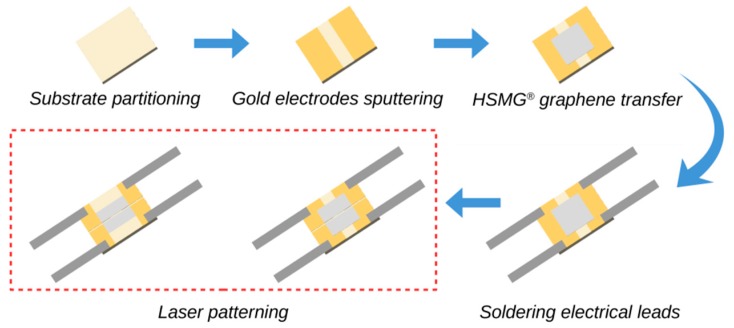
Method of sample preparation. HSMG ^®^: High-strength metallurgical graphene.

All the tested samples were designed according to the same method. The first step in the design process was substrate partitioning (Figure 3). Laser cutting was used to create samples of the desired size (10 × 8 mm). This involved making thin notches (width 80 μm, depth 300 μm) in the ceramic substrate, which could then be broken easily into individual parts. This solution provided a clean surface without defects, which was subsequently used in the next processes: PVD and HSMG^®^ graphene transfer. The well-known PVD method was applied to produce gold electrodes on the ceramic substrate. The rectangular shape of the electrodes (8 × 4 mm) was the effect of the masking process and the PVD method. A modified transfer procedure of HSMG^®^ graphene was used. A thin film of PMMA was used as a graphene-supporting material. Variations of the applied method are commonly used for the transfer of 2D materials (graphene) onto substrates of any kind. Wrinkles and cracks were observed on the graphene after the transfer process, the explanation for which has been discussed in a previous work [63]. It should be noted that the transfer method used does not provide sufficient precision for directly shaping the sensory layer. The repeatability obtained was good but not adequate for sensor production. Heat treatment was applied in a vacuum after the transfer process to improve the adhesion of the layer to the substrate and minimize the stress on the graphene–gold–ceramic junction.

Strips of silver thin foil (35 μm) were soldered to the gold electrodes on the substrate surface to make metallic, elastic electrical leads. Indium soldering was used and the obtained joints were protected against oxidation by using polyurethane resin. Each of the samples had two pairs of the same electrodes, which allowed for the electrical parameters to be measured before and after the laser treatment (Figure 3). Although laser patterning allows for the creation of any shape, only samples with simple shapes were used in the tests. The simple rectangular shapes of the graphene and gold electrodes facilitated a more reliable analysis of the results.

### 2.2. Instrumentation and Measurement Procedure

The areas of the samples with and without laser treatment were studied via microscopic examination and Raman spectroscopy. Due to the nature of graphene, SEM, TEM, and AFM techniques are preferable for the examination of graphene structures, although optical microscopy can also be useful. In our research, the quality of the patterning of the graphene structures was assessed using optical microscopy (Neophot 21, Carl-Zeiss Jena, Germany and SEM (Hitachi S-4200, Hitachi Ltd., Chiyoda-ku, Tokyo, Japan). To obtain good quality SEM images, the vacuum in the microscope chamber was decreased to 100 hPa.

For the graphene quality analysis, a Renishaw inVia Raman spectrometer (Renishaw plc, Gloucestershire, UK) was used. The experiments were carried out using a 532 nm laser with a 50× objective lens (Carl-Zeiss Jena, Germany). The Raman spectra were analyzed at a spectral resolution of 1 cm^−1^ in the spectral range of 1100–3200 cm^−1^. Acquisition times varied between 120 and 200 s (to improve the signal-to-noise ratio). The signals were collected from nine points: zero (in the middle of the laser cutting line) and eight points at distances of 50 μm (points 1 and 5), 60 μm (points 2 and 6), 75 μm (points 3 and 7), and 100 μm (points 4 and 8) from the zero line. The spectra were deconvoluted in Peakfit 4.11 software (Seasolve, Framingham, MA, USA).

**Figure 4:**
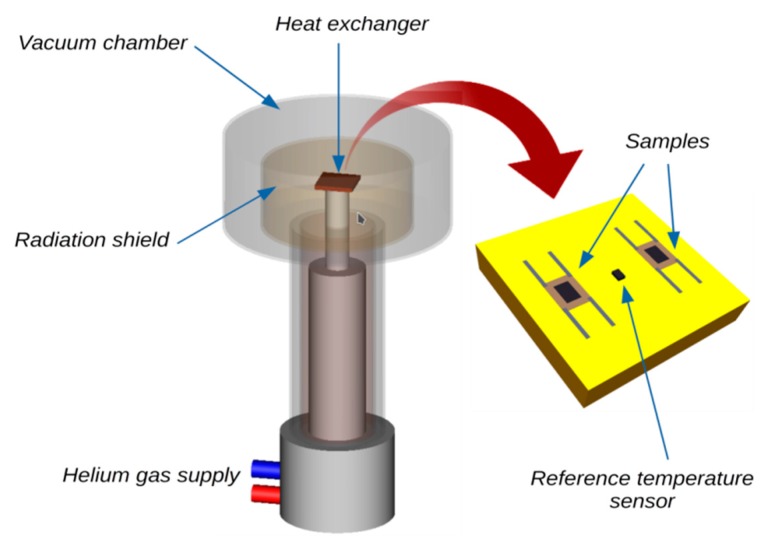
Cryogenic cooling system.

All measurements of the electrical properties of graphene on the ceramic substrate were performed in a helium closed-cycle DE-210 cryostat (Advanced Research Systems, Inc., Macungie, PA, USA). The samples were placed in a vacuum chamber and cyclically cooled from 293 K to 15 K at a rate of about 4 K/min (Figure 4). The tested samples and the reference temperature sensor (DT-670-SD silicon diode Lakeshore Cryotronics Inc., Westerville, OH, USA) were each mounted directly on the massive copper heat exchanger. The entire surface of the sample was fixed to the heat exchanger to eliminate the temperature gradient in the substrate. The massive heat exchanger was mounted directly to the “cold finger” of the cryocooler.

The four-probe method was used to measure the resistance of the samples. Resistance measurements were conducted using a Keysight 34420A Micro-Ohm Meter (Keysight Technologies, Santa Rosa, CA, USA). Two-stage tests of the samples were performed. In the first stage, the electrical properties of the samples were measured, followed by laser patterning. The results showed the parameters of the original, primary samples, with possible defects and impurities resulting from the transfer process. In the second stage, the laser-processed samples were tested using the same method. This measurement procedure enabled the impact of the laser processing on the electrical parameters of the samples to be identified via comparison with the previous results. Additionally, measurements across a wide temperature range enabled the type of electrical conductivity to be identified, as well as the associated phenomena, such as the phonon scattering and defects. These measurements allowed us to observe the interaction of the graphene layer with the ceramic substrate, particularly at cryogenic temperatures, and the effect of the substrate on the parameters of the layer. The results are particularly important for the construction of cryogenic sensors (temperature sensors, Hall sensors, etc.) based on graphene [64].

## 3. Results and Discussion

### 3.1. Laser Patterning of the Graphene Layer

**Figure 5:**
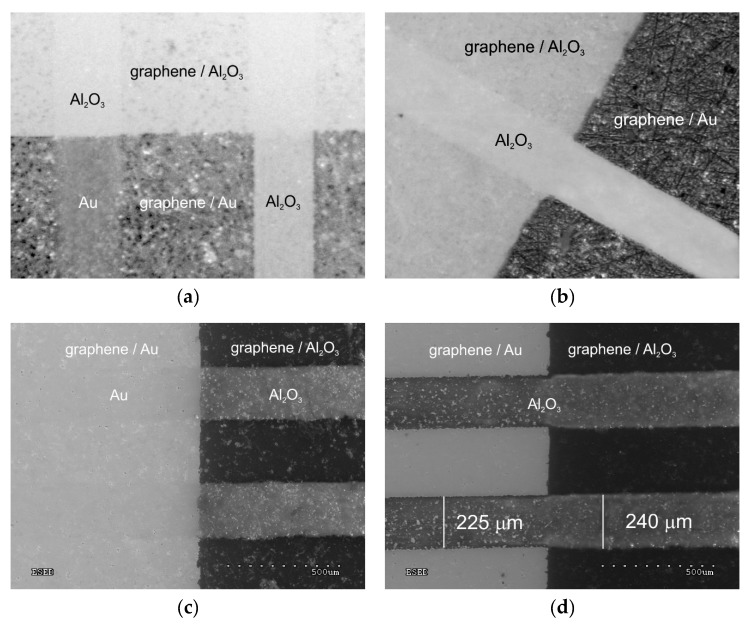
Microscopic pictures of graphene structures (paths) shaped by laser ablation: (**a**,**b**) optical microscopy and (**c**,**d**) SEM.

The influence of the patterning process parameters on the effectiveness of the removal of the graphene layer from the alumina ceramic surface and Au electrodes was examined experimentally. Tests were performed at a constant pulse overlap ratio of 1:3. The effectiveness of graphene ablation was determined using measurements of electrical conductivity on the ablated path. In the absence of electrical conductivity, the possible presence of graphene fragments was additionally investigated using Raman spectroscopy. In our preliminary studies, the highest edge quality was obtained with pulses of the shortest duration t_p_ = 15 ns, repetition frequency 290 kHz, at a scan velocity of 2500 mm/s, and with a hatching of 10 μm. Further research focused on determining the threshold value for the pulse energy. The pulse energies needed to achieve complete ablation were 18 μJ for graphene on alumina ceramic, 24 μJ for graphene on Au electrodes, and 34 μJ for simultaneous ablation of the graphene layer and Au film. Microscopic pictures of the three cases of graphene ablation are shown in Figure 5.

Both optical microscopy (Figure 5a,b) and scanning electron microscopy (Figure 5c,d) confirmed the effective removal of the graphene layer by laser ablation. Using the appropriate pulse energy value (24 µJ), complete ablation of graphene was achieved both from the ceramic substrate and from the Au electrodes (without damaging the gold layer). A greater pulse energy (34 µJ) resulted in both the ablation of graphene from the ceramic substrate and the simultaneous ablation of the graphene and Au electrode layer without damaging the ceramic substrate. These results indicate the possibility of shaping graphene structures at every stage of the production sensors, while also trimming the resistance value of the sensory layer. The shaped structures exhibited an acceptable edge quality, with small shell-shaped irregularities due to the diameter of the laser beam spot (26 µm) and the pulse overlap ratio of 1:3 (Figure 2).

In the microscopic images (Figure 5b,d), there is a noticeable change in the width of the ablation path created by the pulses of energy providing the simultaneous removal of graphene and the Au layer. In this area on the Al_2_O_3_ substrate, the ablated path width was ≈240 μm. In the area on the Au layer, the path width was ≈225 μm. The path width was greater than the assumed value (200 μm) due to the laser micro-machining method used (pulse over pulse, Figure 2) and the Gaussian distribution of the power density in the cross-section of the laser beam. The Gaussian beam diameter d_0_ (26 μm in our case) was defined at the intensity level I_max_/e^2^, which means in practice that the laser beam–surface interaction occurred on an area with a larger diameter. In particular, in thin-layer micromachining, the diameter of the ablation area is greater than d_0_. The reduction in path width on the Au layer area may be explained similarly. The overlapping of pulses “inside” the ablation path provided an averaging of the absorbed energy (Figure 2). The effects of the pulse trains at the edges of the path reflected the uneven (Gaussian) distribution of surface power density. The ablation of the graphene layer on the Al_2_O_3_ substrate (thickness of several nanometers) occurred at a lower threshold value of absorbed energy than the ablation of the Au layer (several hundred nanometers). Changes in the sizes of the paths were more noticeable when they had smaller widths.

### 3.2. Raman Spectroscopy Results

**Figure 6:**
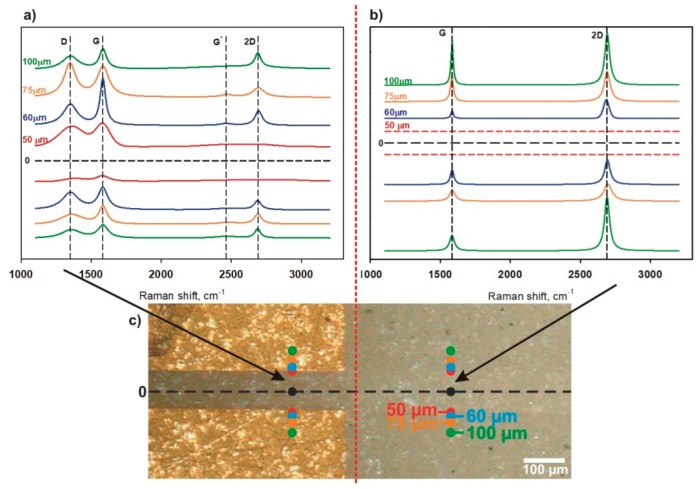
Raman spectra of high strength metallurgical graphene (HSMG^®^) after laser machining: (**a**) Raman spectrum of laser irradiated graphene on a Au/Al_2_O_3_ substrate, (**b**) the Raman spectrum of laser-irradiated graphene on Al_2_O_3_, and (**c**) sample marked with points of acquisition of the Raman spectra.

The Raman analysis of a laser-ablated path is presented in Figure 6. The laser ablation of graphene on both the Al_2_O_3_ substrate and Au layer was effective. This was confirmed by the absence of graphene and other carbon forms in the middle area of the path (below ± 50 µm from the centerline) (Figure 6). The first graphene peaks on the Al_2_O_3_ substrate appeared at the points lying 60 µm above and below the centerline (Figure 6b), where G and 2D bands were visible. These Raman shifts are typical for graphene and multilayered graphene. The Raman spectrum of graphene on the Au layer looked different. Additional peaks, such as D and G* were seen (Figure 6a) at points beyond the middle area (over ± 50 µm). Raman spectra obtained near the edges of the cutting line when graphene was removed from gold are characteristic for graphene oxide and reduced graphene oxide. The characteristic values of the ID/IG band intensity ratios were observed [65,66,67]. The same laser treatment parameters were used for the ablation of graphene on Au and Al_2_O_3_, but the width of the path was different (as mentioned above). This may indicate that some portion of laser energy was used in the oxidation process of graphene. To determine the degree of oxidation, XPS tests would be required; however, in the presented work, further considerations about the oxidation degree of graphene were omitted.

The results of the Raman spectroscopy revealed G and 2D peaks that are typical for graphene structures, with a higher intensity corresponding to the 2D band. The intensity relation of IG/I2D indicates that the investigated graphene was a multilayer structure. According to the literature, an IG/I2D relation of ≈0.2–0.8 indicates one to six graphene layers [68]. In our case, the IG/I2D ratio was between 0.3 and 0.8. Additional peaks for graphene (D, D’, G*) were not observed on the Al_2_O_3_ substrate. The absence of additional peaks suggests that the graphene structure was not disordered. The 2D and G band intensities did not depend on the distance from the center of the ablation path. Therefore, it can be concluded that the graphene was not affected by laser irradiation. 

In the case of the Al_2_O_3_ substrate, the paths after the laser cutting were graphene-free at distances of 50 μm above and below the centerline, while a peak D could be observed in the Raman spectrum for the laser-ablated graphene on the Au layer. The appearance of the D band is related to defects occurring in the graphene structure [68,69].

The Raman spectra showed different quantities of defects, expressed by the ID/IG intensity ratio (values 0.5 to 1.1). The intensity ratio depends on the distance from the centerline but the number of defects was random (Figure 6a). Our observations seem to be similar to those reported in the literature [68,69,70,71] but the reason for the occurrence of defects in our studies was different. It should be noted that ablation using nanosecond pulses is to a significant degree thermal in nature. The thermal properties of the substrate have a significant impact on the ablation of the graphene layer. The results presented in the literature relate mainly to the laser treatment of graphene on SiO_2_/Si substrates, where local heat accumulation is observed, which is associated with the thermal properties of the substrate. The thermal conductivity of the substrate plays an especially important role in the thermal processes. It should be noted that the thermal conductivity of SiO_2_/Si is about twenty times lower than that of Al_2_O_3_, while the specific heat of both is similar, which determines the dynamics of the thermal processes. The thermal parameters of the graphene layer are insignificant in this process because the layer is very thin; therefore, the longitudinal heat flux in the layer is negligible. The same thermal processes occur in the Au layer (thickness around 150 nm). This means that the substrate is generally responsible for heat accumulation and dissipation during the laser treatment process. Disturbances to the graphene structure occurring after laser treatment near the edges of the ablation area may also be the result of the deposition of Au decay products on the graphene. 

### 3.3. Temperature Dependence of Resistance

**Figure 7:**
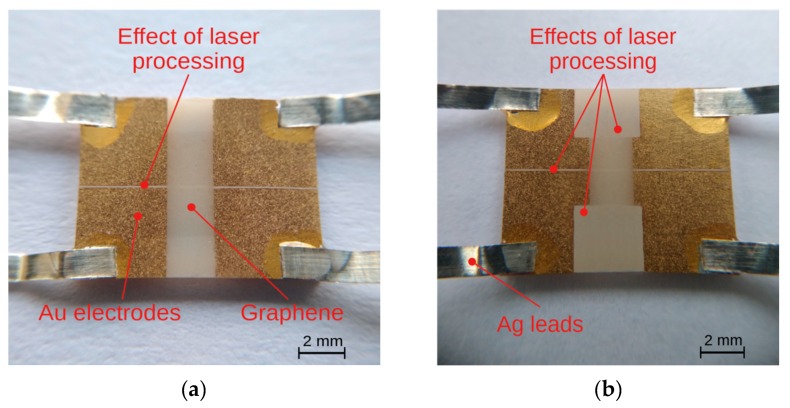
Sample after laser patterning: (**a**) the HSMG^®^ graphene and gold electrodes were cut and (**b**) the HSMG^®^ graphene and gold electrodes were shaped.

**Figure 8:**
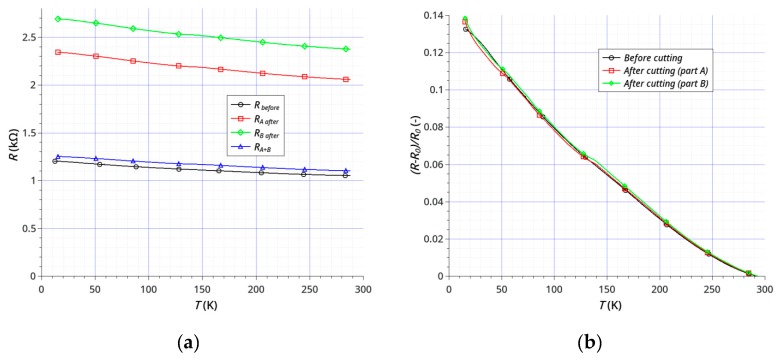
Temperature dependence of the resistance of the sample before and after laser cutting: (**a**) resistance *R* (kΩ) of sample and (**b**) relative change in the sample resistance (*R_0_*—initial resistance of the sample at *T*_init_ = 293 K).

**Figure 9:**
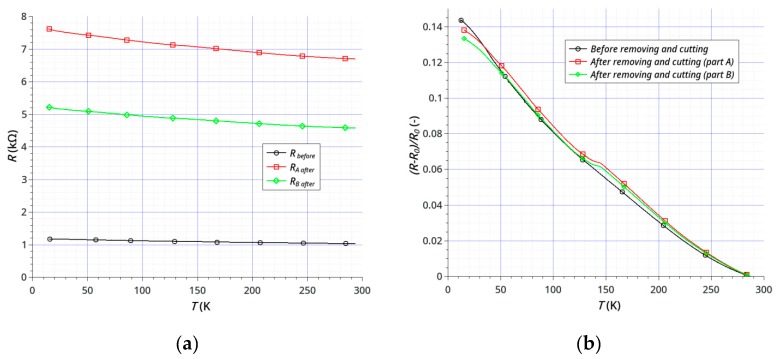
Temperature dependence of the resistance of the sample before and after laser cutting and reducing: (**a**) resistance *R* (kΩ) of the sample and (**b**) relative changes in the sample resistance (*R_0_*—initial resistance of the sample at *T*_init_ = 293 K).

The dependence of sample resistance on temperature was investigated using samples before and after the laser treatment. The treatment consisted of cutting (Figure 7a) and cutting with the removal of parts of the graphene and gold layers (Figure 7b). The main purpose was to examine the possibility of direct laser shaping of the sensors by cutting and (or) removal of graphene layers either from ceramics or from an electrode layer (Au), or removal of graphene and electrode simultaneously. We have shown that it is possible to shape simple geometries without any damage to either the graphene or gold. Figure 8 and Figure 9 show the results of testing two samples before and after laser processing. The temperature dependence of the resistance is shown in Figure 8a and Figure 9a, and the change in resistance related to the sample resistance at 293K is shown in Figure 8b and Figure 9b. Both the active graphene layer and the gold electrodes were cut with a cutting width not exceeding 120 μm.

**Figure 10:**
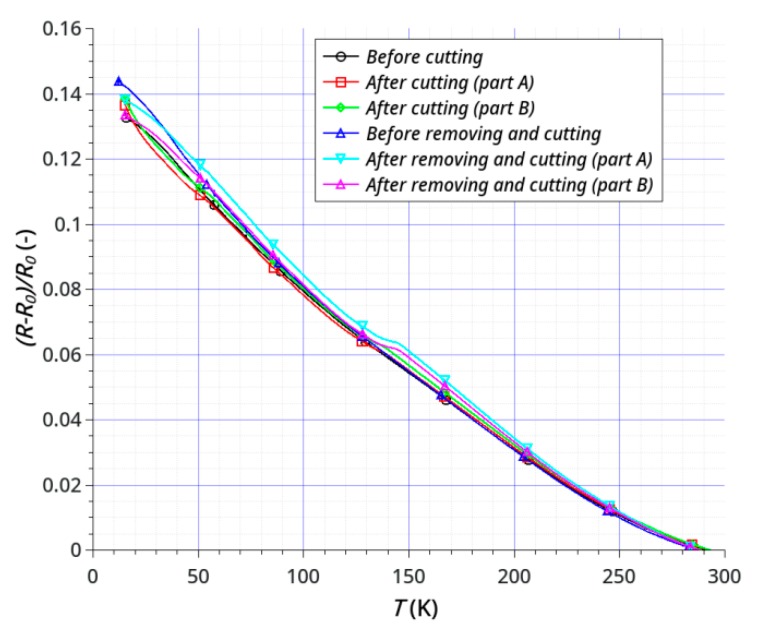
Relative changes in the sample resistances before and after the laser treatment (*R_0_*—initial resistance of the sample at *T*_init_ = 293 K).

The main goal of our research was to achieve the effective and non-destructive removal of graphene from Al_2_O_3_ and the effective shaping or partitioning of graphene on the Au layer. Our results show that it is possible to remove graphene from the Au layer on an Al_2_O_3_ substrate without damaging the Au layer (Figure 5). The results of electrical studies confirmed the effectiveness of our method (Figure 8 and Figure 9). The effects of temperature on the samples resistances showed a continuous, close-to-linear decrease in the resistance across the whole range of 15–293 K. Figure 8 and Figure 9 present the results of testing two samples before and after laser treatment. The graphene layer and Au electrodes on the first sample (Figure 7a) was divided (cut) into two parts of similar dimensions. The sample resistance before cutting was about 1.05 kΩ at 293 K. The resistances of each of the elements after the division were 2.06 kΩ and 2.38 kΩ, respectively. Figure 8b shows the relative change in resistance before and after the split. The nature of the sample resistance changes before and after the division was the same. This confirms that there was no additional degradation of the graphene layer on the ceramic substrate between the gold electrodes after laser processing. The continuity of the layer and the lack of defects in this area also confirmed the results of the Raman spectroscopy (Figure 6b). This is a very important result because the initial parameters of the graphene layer on ceramics did not change. On this basis, we can assume that the usage of this method will be useful for the production or trimming of sensors. The calculated value of resistance of the parallel-connected parts obtained after cutting (*R_A+B_*) was slightly higher than the initial resistance of the sample (*R_before_*). This was the effect of a small loss of material as a result of the laser processing. The second studied sample, like the first, was divided into two parts and the sizes of the graphene layer and gold electrodes were also reduced (Figure 7b). The purpose of this study was to show that removing a significant area of the layers would not change their electrical properties (excluding resistance). The initial resistance of the sample before processing was 1.03 kΩ. The graphene layer and electrodes were partially narrowed, with different widths obtained. The resistances of each of the elements after the division and the reduction in dimensions were 4.58 kΩ and 6.69 kΩ. It should be noted that the relative changes in the resistance of both types of samples before and after laser treatment were very similar (Figure 8b and Figure 9b). This confirms the absence of significant degradation of the graphene layer in the area between the gold electrodes. The differences did not exceed 2% (Figure 10). This is very important because this is the active area that is responsible for the parameters of sensors. The significant convergence of the results obtained confirms the local nature of the laser ablation of the layers (Figure 10). For all studied samples before and after the laser processing, the negative temperature coefficient of resistance (TCR) was close to −5.5 × 10^−4^ K^−1^ and slightly increased at low temperatures below 60 K. We have observed a similar effect in our previous studies [64]. A slight degradation of the graphene in the area of the gold electrodes was less important than good contact between the layers (Figure 6a). A sustainable and ohmic electrical connection across a wide temperature range is especially crucial in the design of most types of sensors. Moreover, the use of a gold layer allows for the soldering of a silver foil to make elastic, metallic electrical leads for cryogenic applications.

It should be noted that all the studied samples were tested in a vacuum environment but the pressure inside the cryostat was not constant or stabilized. Changes in pressure are a natural result of changes in the temperature of the rest gases and it was impossible to stabilize the pressure inside the cryostat. To minimize and standardize the impact of the vacuum on the parameters of the samples (opened samples without encapsulation), they were placed in a vacuum 24 h before testing. Our previous studies showed that samples based on HSMG^®^ graphene exhibit a slight sensitivity to pressure changes. The observed changes in resistance stabilize below 100 mTorr, and a longer duration in a vacuum (over 12 h) eliminated the impact of pressure changes during the cryogenic tests. An encapsulation process was therefore necessary to obtain samples with repeatable and stable parameters. Effective technology is currently under development.

## 4. Conclusions

The most important advantage of the presented method is the ability to simultaneously shape the graphene and Au layers on a ceramic substrate without causing significant damage, especially on the graphene layer in the sensory area, i.e., graphene on ceramics. The usage of laser processing ensures the high speed and repeatability of the process and allows for the shaping of any simple geometries without the masking process. All presented results (optical microscopy, SEM, Raman spectroscopy, and electrical properties) confirmed the effectiveness of the proposed method. In addition, the use of a ceramic substrate ensures the optimal thermal parameters of the sensor, i.e., high thermal conductivity, low thermal expansion, and high electrical resistivity. The local nature of the laser ablation process provided good shape mapping and did not cause significant defects. However, the sample preparation method required miniaturization and encapsulation of the layer to stabilize the parameters. A drawback of the presented solution is that the temperature range is limited to cryogenic temperatures. The limitation is directly related to the electrode assembly technique (indium alloy soldering), not to the properties of the graphene layer and ceramic substrate. Studies in the range above room temperature require changes in the sample preparation method mentioned earlier. The linear relationship *R*(*T*) is important for the design of the signal conditioning system; however, a small TCR value can be a source of additional errors in the potential temperature sensor. In our opinion, the presented method has notable application potential and can be used for both the production and trimming of sensors based on graphene on a ceramic substrate.
